# Nature Play and Fundamental Movement Skills Training Programs Improve Childcare Educator Supportive Physical Activity Behavior

**DOI:** 10.3390/ijerph17010223

**Published:** 2019-12-27

**Authors:** Pulan Bai, Ashleigh Thornton, Leanne Lester, Jasper Schipperijn, Gina Trapp, Bryan Boruff, Michelle Ng, Elizabeth Wenden, Hayley Christian

**Affiliations:** 1 School of Population and Global Health, The University of Western Australia, Crawley, Western Australia 6009, Australia; gina.trapp@telethonkids.org.au (G.T.); elizabeth.wenden@telethonkids.org.au (E.W.); hayley.christian@telethonkids.org.au (H.C.); 2 Telethon Kids Institute, University of Western Australia, Nedlands, Western Australia 6009, Australia; michelle.ng@telethonkids.org.au; 3 School of Human Sciences, The University of Western Australia, Crawley, Western Australia 6009, Australia; ashleigh.thornton@uwa.edu.au (A.T.); leanne.lester@uwa.edu.au (L.L.); 4 Department of Sports Science and Clinical Biomechanics, University of Southern Denmark, 5230 Odense, Denmark; jschipperijn@health.sdu.dk; 5 School of Agriculture and Environment, The University of Western Australia, Crawley, Western Australia 6009, Australia; bryan.boruff@uwa.edu.au

**Keywords:** Early childhood education, professional development, childcare, preschool, physical activity behavior

## Abstract

Background: Physical activity professional development programs for Early Childhood Education and Care (ECEC) educators are a viable strategy for improving preschool children’s physical activity behavior. This pilot intervention evaluated the effectiveness of ‘nature play’ and ‘fundamental movement skills’ (FMS) professional development programs on ECEC educators’ practices on physical activity. Methods: 148 ECEC educators from 20 ECEC centers took part in either the Nature play or FMS professional development programs in Perth, Western Australia. Educators self-reported their physical activity related practices at baseline and three months post-professional development training, using established items. Wilcoxon’s test and adjusted models using Mann–Whitney U tests were run at the individual educator level to examine the change between baseline and post-professional development educator physical activity behavior. Results: Educators’ self-efficacy to engage children to be active significantly increased in both the Nature play and FMS professional development programs (*p* < 0.05). In the Nature play professional development program, ECEC educators’ perceived time set aside for children to participate in nature-based play increased by 9.2%, and their perceived behavioral control for supporting general and nature-based play activities for preschool children increased by 5% and 10.3%, respectively (*p* < 0.05). However, these results were no longer significant after adjusting for educator socio-demographics. Conclusion: Both the Nature play and FMS professional development programs were effective in improving educators’ self-efficacy to engage children to be active in Nature play or FMS activities. Larger pragmatic trials are required to confirm the impact of these professional development programs on educator perceived physical activity behavior.

## 1. Introduction

Physical activity is vital for preschool children and its benefits include developing healthier bones, maintaining a healthy weight, and improving cognitive development [1]. The Australian 24-h movement guidelines for the early years recommend children aged 1–5 years accumulate at least 180 min of physical activity per day for health and developmental benefit [2]. However, a significant proportion of children do not meet the recommended 180 min of physical activity per day. Objective measurement of Australian preschool children’s physical activity shows that only a third of children meet physical activity recommendations [3]. In Canada, 62% of preschool children meet physical activity recommendations [4] and in Belgium less than 20% of preschool aged children meet the recommended amount of physical activity per day [5]. Therefore, interventions to promote physical activity in the early years is paramount.

ECEC centers are an important setting for physical activity promotion as most children aged 2–5 years attend an ECEC center; across Organization for Economic Co-operation and Development (OECD) countries more than 80% of children aged 3–5 are enrolled in childcare or primary school [6]. Considering that many preschool children spend a large proportion of their time in the ECEC environment [7], professional development programs for ECEC educators may be effective in promoting positive physical activity behaviors within the ECEC setting. Research has shown that physical activity based professional development programs may help educators to support and promote physical activity in ECEC centers. For example, Tucker et al. [8] reported educators felt that additional training and resources are important to reduce sedentary behavior and increase physical activity in preschool children. Furthermore, a review by Egert et al. [9] suggested that quality improvement via ECEC in-service training is a key mechanism to accelerate the development of young children. Thus, educator professional development programs with a focus on physical activity may be an effective and sustainable strategy for improving preschool children’s physical activity in ECEC centers.

In Australia, various types of formal ECEC exist for different family needs. These include long day care, occasional care, family day care, and in-home care services [10]. Long day care is the most common form of formal ECEC [11]. Long day care provides all-day or part-time care for working families and offers developmental programs for large groups of children in line with the Australian Early Years Learning Framework [12]. This makes long day care (referred from here on as ECEC) the most suitable setting to implement physical activity professional development programs.

Physical activity professional development programs for preschool educators can promote active play and/or be focused on a specific child development outcome, such as fundamental movement skills (FMS). FMS include locomotor, object control, and stability and contribute to children’s physical, social, and cognitive development [13]. Early childhood is a critical period for FMS development and mastery, and there is evidence that preschool children’s fundamental movement skills are associated with physical activity participation [14]. These findings support the importance of development of FMS in childhood, suggesting that effective educator-based FMS professional development programs may be a sustainable way to effectively refine these skills in preschool children so that they can grow into physically confident, active and healthy children. FMS professional development programs in ECEC environments have been shown to have a positive effect on preschool children. For example, Jones et al. [15] found small to medium effect sizes in favor of the FMS professional development intervention group compared to the control group. Despite the importance of FMS, the impact of FMS interventions on ECEC educators appears to be understudied [16]. It is therefore vital for studies to evaluate the impact of FMS professional development programs on educator physical activity practices.

Nature play professional development programs focus on promoting outdoor nature-based active play in the ECEC environment by interacting with natural elements. Natural elements (e.g., trees, shrubs, sand, and water) in the ECEC outdoor play environment can offer more play opportunities for children and increase physical activity engagement [17]. Natural outdoor experiences in young children are associated with the development of sense of identity, autonomy, psychological resilience, self-regulation, and gross motor skills [18]. Nature play professional development programs empower educators to use the space and natural resources available to them in interactions with children, and provide children the opportunity to actively play and build strong connections with the natural world [19]. There appears to be no studies investigating the impact of nature-based play or outdoor nature based professional development on ECEC educators’ physical activity practices.

There is a clear need to investigate the impact of Nature play and FMS professional development programs to see if these initiatives can improve ECEC educator physical activity behaviors. The aim of this study was to pilot test if Nature play and FMS professional development programs improve ECEC educator behavior and practices around preschool children’s physical activity in ECEC centers. The secondary aim of this study is to investigate the effect of educators’ level of qualification, age, experience in the sector, and working hours on the intervention effect. We hypothesized that both professional development programs would improve educator physical activity-related behavior and practices overall, but due to the different content and techniques taught in the two professional development programs the effects may vary across the different educator physical activity behavior and practices measured. We also hypothesized that the intervention effect would be less for those educators who are older, more highly qualified, work longer hours and have longer work experience. This is because these educators may have high baseline levels of physical activity-related behaviors and practices causing the intervention effect to be minimal.

## 2. Materials and Methods 

### 2.1. Study Design and Procedure

**Figure 1:**
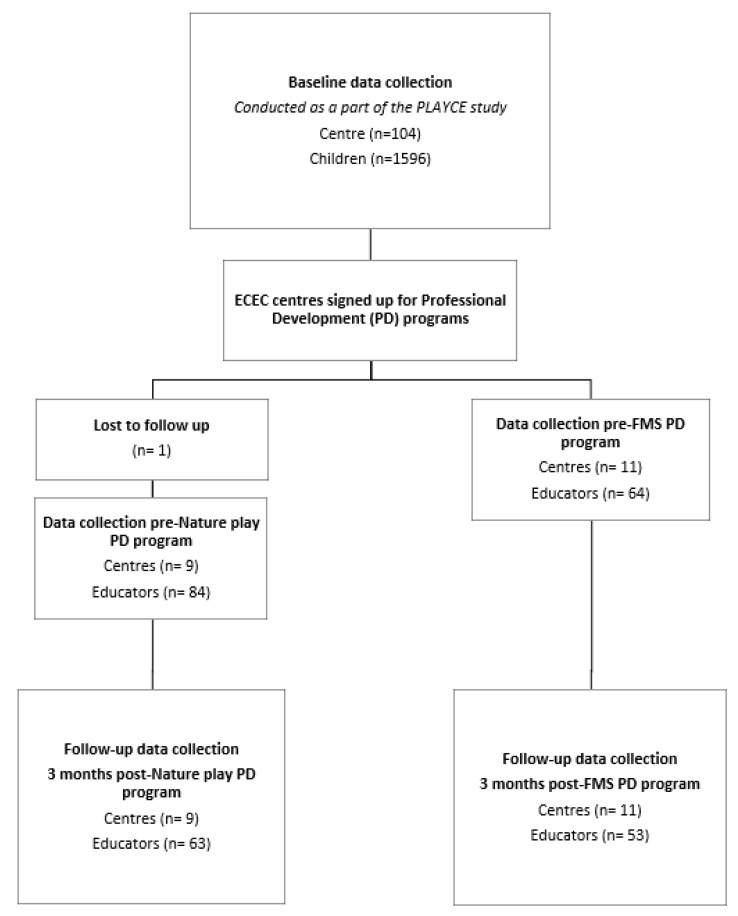
Overview of the study design and recruitment process. ECEC: Early Childhood Education and Care; PLAYCE: Play Spaces and Environments for Children’s Physical Activity; FMS: Fundamental movement skills.

This study was a sub-study of the larger Play Spaces and Environments for Children’s Physical Activity (PLAYCE) study. The PLAYCE study was a three-year program (2015–2017) investigating the effect of the ECEC environment on physical activity in the early years. It was a cross-sectional observational study which collected data from 1596 preschool children aged 2–5 years from 104 early ECEC centers in metropolitan Perth, Western Australia. The protocol for the PLAYCE study has been published [20]. The current study aimed to pilot test the feasibility and impact of two physical activity professional development programs for ECEC educators. An overview of the study design is shown in Figure 1.

### 2.2. Participants

All centers that took part in the PLAYCE study were invited to enroll in either a FMS or an outdoor nature-based play (nature play) professional development program. To enroll in the professional development program ECEC centers were asked to sign up and pay for the professional development program. 11 ECEC centers (84 educators) enrolled and completed the nature play professional development program and nine ECEC centers (64 educators) enrolled and completed the FMS professional development program. 

Educators from the enrolled ECEC centers completed a survey prior to the professional development training and again three months after the professional development training; 63 educators from the Nature play professional development program and 53 ECEC educators from the FMS professional development program completed the survey post professional development (Figure 1). The surveys were administered face to face and took 10 min to complete.

#### Eligibility Criteria and Ethical Consideration

Eligible centers were long day care centers located within the metropolitan Perth, Western Australia. All participating ECEC centers signed up to either of the professional development programs voluntarily. Centers were eligible to take part if they had their baseline data collected in the last three months as part of the larger PLAYCE study [20]. Once a center had signed up to one of the professional development programs, all ECEC educators working at that center were asked to complete the program. An ethics amendment to the PLAYCE Study for this pilot study was granted by The University of Western Australia Human Research Ethics Committee (#RA/4/1/7417). All educators in this study provided written consent prior to the start of the professional development programs. All participants were given the right to withdraw from the study at any time.

### 2.3. Professional Development Programs

#### 2.3.1. The Fundamental Movement Skills Professional Development Program

The FMS professional development program was developed in collaboration with the School of Human Sciences, University of Western Australia [21]. It aimed to provide ECEC educators with an understanding of the processes of motor development; specifically the development and training of FMS and motor proficiency, and how to engage children in more physical activity whilst at ECEC centers. Practical information, such as skill development activities and strategies for creating action plans were provided to ECEC educators to encourage sustainable physical activity promotion in their centers. There was a maximum of six hours of contact time: A two-hour shadowing exercise two weeks prior to the workshop in which the FMS specialist went into the center to observe educator practices related to physical activity; a two-hour personalized professional development workshop for all educators from the center; and another two-hour shadowing exercise two weeks after the workshop to observe educator practices and provide feedback.

#### 2.3.2. The Nature Play Professional Development Program

The Nature Play professional development program was developed in collaboration with Nature Play Western Australia [19]. This professional development program aimed to provide educators with an understanding of the benefits of outdoor nature-based play and how to encourage outdoor play in the ECEC setting in order to increase the time children spend in unstructured outdoor play. The focus of this program was to provide and support educators with practical ways of providing opportunities for children to connect with nature on a regular basis. It contained strategies and prompts for educators that can be applied in the ECEC setting with simple preparation and minimal materials. This program contained approximately four hours of contact time: A one-hour shadowing exercise to observe ECEC educator practices and the ECEC environment to personalize the workshop; a one-hour incursion with a group of children and educators to demonstrate how natural materials can be used to support children’s play and learning using five different natural elements (sand, water, a tray, containers and spoons, stone surface) followed by a two-hour professional development workshop with all educators from the center.

ECEC educators in both the FMS and Nature Play professional development programs were asked to complete a survey before the program, and then again three months after the program. Full details on the survey measures are described below.

### 2.4. Survey Measures

Each subscale in the survey was adapted from a validated survey instrument. The survey included two sections; the first section focused on general physical activity and the second focused on the specific professional development program educators participated in (i.e., FMS or nature play). A copy of the survey is available from the senior (last) author on request.

#### 2.4.1. Importance of Physical Activity to Educators

The perceived importance of physical activity sub-scale was adapted from the Intrinsic Motivation Inventory (IMI) which is a well-established tool used to assess participants’ subjective experience related to a target activity [22]. Educators reported if they agreed with a) “It is really important to me that I am physically active regularly”, b) “It is really important to me that children at our center are physically active regularly” and c) “It is really important to me that I provide children with opportunities to be physically active regularly”. Items were evaluated on a seven-point Likert scale ranging from 1 (strongly disagree) to 7 (strongly agree), and an average score created according to the IMI guide [22]. In past research the adapted IMI was shown to have acceptable pre-test (α = 0.67) and post-test (α = 0.86 and 0.89) internal consistency. 

#### 2.4.2. Perceived Behavioral Control Over Child Physical Activity Support

Items from the perceived behavioral control over child physical activity support subscale developed by Rhodes and colleagues [23] were used. This subscale has acceptable reliability (α = 0.85) [23]. Educators were asked how often they a) “encourage children at the center to participate in physical activity”, b) “participate in physical activity with children at the center”, and c) “provide equipment at the center for children to participate in physical activity”. Responses were scored as never or rarely; about once or twice a month; 1–2 times per week; most days or daily. Scoring followed the method outlined by Rhodes et al. [23]; scores were averaged for the three items dichotomized as low (<3.50) or high (>3.49) frequency. Variables were created for general physical activity as well as for FMS and nature-based play specifically.

#### 2.4.3. Perceived Regulation of Supportive Behaviors for Physical Activities

Rhodes and colleagues’ [23] behavioral regulation of support behaviors subscale was adapted to measure educator perceived regulation of supportive behaviors for physical activity, as well as for FMS and nature-based play specifically. This subscale has acceptable reliability (α = 0.79) [23]. Educators were asked how often they a) “look for information or opportunities to facilitate children’s engagement in physical activity”, b) “make plans in advance for supporting children’s participation in physical activity at the center”, c) “Set goals for how much physical activity children will get while at the center”, and d) “Monitor (e.g., keep records of) how much physical activity children are participating in at the center”. We also asked how often educators “set aside time at the center specifically for children to participate in physical activity”. Responses were scored as never or rarely; about once or twice a month; 1–2 times per week; most days or daily. Scoring followed the method by Rhodes et al. [23] and was the same as Perceived Behavioral Control Over Physical Activity. 

#### 2.4.4. Educator Self-Efficacy to Engage Children to be Active

Six items were adapted from Jackson and colleagues’ [24] nine item “other-efficacy” scale to measure educator’s self-efficacy to engage children to be active. The internal reliability estimate for this subscale was shown to be acceptable (α = 0.90) [24]. Educators were asked how confident they were in their ability to: (a) “Motivate the children at their center to be physically active regularly”, (b) “Motivate the children at their center to join in even during hard or unfamiliar physical activities”, (c) “Make sure that time spent being physically active at their center was fun and enjoyable for the children”, (d) “Provide a variety of activities that make physical activity interesting for children”, (e) “Provide individualized attention to all of the children they supervise during physical activity”, and (f) “Provide helpful and instructive feedback to all of the children they supervise during physical activity”. Responses ranged from 1 (no confidence at all) to 5 (complete confidence). Scoring followed the Jackson et al. [24] method in which a mean score was created for the six items.

#### 2.4.5. Educator Motivation to Engage Children in Physical Activities

The work tasks motivation scale for teachers consists of 15 items per task and has overall acceptable internal consistency (α > 0.70) [25]. We adapted 10 items to create a measure of educator motivation to engage children in physical activities. Educators were asked what their reasons were for engaging children in physical activity. Questions included: (a) “Because it is pleasant to carry out physical activity tasks with the children”, (b) “Because I find it interesting”, (c) “Because it is important for me to carry out”, (d) “Because I find it important for the development of the children I work with”, (e) “Because I would feel guilty not doing it”, (f) “To not feel bad if I don’t do it”, (g) “Because the center obliges me to do it”, (h) “Because I’m paid to do it”, (i) “I don’t know, I don’t always see the relevance of doing it”, and (j) “I used to know why I did it, but I don’t see the reason anymore”. Items were grouped into “Intrinsic Motivation” (items, a, b), “Identified Motivation” (items c, d), “Introjected Regulation” (items e, f), “External regulation” (items g, h), and “Amotivation” (items i, j) sub-scales. Responses ranged from 1 (not at all for this reason) to 7 (exactly for this reason). Scoring of this subscale followed the work tasks motivation scale for teachers method: a mean score was created for each of the three sub-scales [25].

#### 2.4.6. Barriers Around Providing Physical Activities

Existing items developed by Kulinna et al. [26] were used to measure educators perceived barriers to providing physical activity opportunities to children in ECEC. Educators were asked how true they thought the following statements were: (a) “Children at my center do NOT enjoy being physically active”, (b) “There is not enough space for children to be physically active”, (c) “The weather prevents children being physically active”, (d) “We do not have time to provide physical activity opportunities throughout the day because of other commitments”, (e) “We do not have the equipment we need to help children be physically active”, (f) “Other educators at our center do not value physical activity”, (g) “The parents at our center do not place a high priority on us helping their children to be physically active”, and (h) “Providing opportunities to be physically active throughout the day does not seem to be a priority in this center”. Responses ranged from 1 (Not at all true) to 7 (Very true). For each item, scores <3 were coded as 0 and scores ≥3 were coded as 1. Scoring for this subscale followed the original subscale and the percentage of endorsement rate (percentage that responded “1”) was calculated.

### 2.5. Statistical Analyses

SPSS V22.0 was used to analyze data. Unadjusted and adjusted models were run at the individual educator level to examine the change between baseline and post-professional development educator behavior. Wilcoxon’s tests were performed to identify significant differences in survey scores before and after the Nature play and FMS professional development programs in the unadjusted models. Normality was tested and within group and between group differences at baseline and post-professional development were assessed using the Mann–Whitney U test. We then ran the repeated measures general linear models again controlling for one socio-demographic factor (educator age, qualification, experience in the ECEC sector and working hours per week at the center) at a time to see which had the most impact on the association. 

## 3. Results

At baseline the mean age of educators was 37.0 (± Standard Deviation (SD) 9.4) in the nature play professional development program and 35.0 (± SD 9.6) in the FMS professional development program. On average educators in the Nature play professional development program had 10.9 years of experience working in ECEC and educators in the FMS professional development program on average had 8.3 years of experience (Table 1). The majority of educators had a diploma qualification (Nature play program = 50.0%, FMS program = 40.6%). There were no significant socio-demographic differences for age, experience in ECEC sector, working hours per week at the center, and highest education level within the Nature play professional development group nor the FMS professional development group (Table 1). No significant differences in educator characteristics were detected between the two professional development programs, as well as between those who did and did not completed the follow up survey in both professional development programs. 73.8% of educators in the Nature play program and 53.9% from the FMS program completed both baseline and post-professional development surveys.

In the unadjusted model, educators’ self-efficacy to engage children to be active in nature-based play or FMS activities significantly increased by 0.33 in the Nature play group (*p* < 0.05) and by 0.26 in the FMS group (*p* < 0.05) on a 5-point Likert scale after the professional development.

In the nature play group, after the professional development the number of barriers around providing physical activities decreased significantly by 0.08 (*p* < 0.05) on a 7-point Likert scale. ECEC educators perceived behavioral control over child physical activity support increased by 5% (*p* < 0.05) for general physical activities and increased by 10.3% (*p* < 0.05) for nature-based play activities after the professional development. ECEC educator perceived time set aside for children to participate in nature-based play increased by 9.2% after the professional development. In the FMS group, the number of barriers around providing physical activities increased significantly by 0.13 (*p* < 0.05) on a 7-point Likert scale after the professional development program.

After adjusting for educator’s baseline age, qualification, experience in the sector and working hours per week at the center, there were no significant differences between baseline and three months post professional development for any of the variables (Table 2).

For our secondary aim we hypothesized that the intervention effect would be less for those educators who were older, more qualified, had more experience in the ECEC sector and who worked longer hours. In the nature play program, educator qualification had a small but significant positive influence on perceived behavioral control over child physical activity support for children’s nature-based play activities (Table 3; ηp2 = 0.05, *p* < 0.05). In the FMS group, experience in the sector also had a small but significant impact on barriers around providing physical activities (ηp2 = 0.04, *p* < 0.05). In the nature play group, experience in the sector also impacted the effect of the intervention on educators reported intrinsic motivation for providing physical activities (ηp2 = 0.25, *p* < 0.05). Working hours per week at the center had a significant influence on the intervention effect of perceived behavioral control over support for children’s FMS activities (ηp2 = 0.13, *p* < 0.05). 

## 4. Discussion

We hypothesized that both the Nature play and FMS professional development programs would improve educators’ physical activity related behavior and practices for children in their care. We also hypothesized that each professional development program would impact different aspects of educator physical activity related behavior and practices due to the different material and techniques taught in the two programs.

In our unadjusted models, ECEC educators from both professional development programs reported significant improvement in self-efficacy to engage children to be active in nature play or FMS activities. This result supports findings by Jones et al. [15], which showed that after training, educators self-efficacy around supporting children’s FMS improved in the intervention group compared with the control group. Moreover, in school teachers there is evidence that professional development programs that improve teachers self-efficacy to provide physical education for children has a positive impact on children’s physical activity [27]. Bandura defines self-efficacy as a person’s beliefs about their ability to produce designated levels of performance for a certain task [28]. Self-efficacy can support desirable behavioral outcomes that lead to increased performance in that task [28]. Overall ECEC educators in both programs were more confident in their ability to motivate children to be active, to ensure that the activities were fun and interesting, and to provide individualized attention and feedback to children during nature-based play or FMS activities.

ECEC educators in the Nature play program reported a significant improvement in their perceptions of control over support for children’s physical activity and nature-based play activities specifically. Educators in the Nature play group also reported that they set aside more time for children to participate in physical activity and perceived that there were fewer barriers around providing physical activities. However, educators in the FMS group reported more barriers. This may be due to the different material and techniques taught in the two professional development programs. Both programs included a 2-h face-to-face evening training workshop. The nature play professional development program also included an in center incursion with one group of children to show ECEC educators how natural materials can be used to support children’s play and learning, this may have helped educators to identify more ways to encourage physical activity in children using natural resources, thus educators perceived that there were fewer barriers around providing physical activities. In contrast the FMS workshop included information on identifying barriers in their center and thus, ECEC educators may have been more aware of potential barriers after the professional development program. Therefore, educators seeking to implement FMS development in their center may need additional support and resources after taking part in professional development programs to help overcome perceived barriers.

ECEC educator physical activity supportive behaviors are important because preschool children spend a significant amount of their time with educators whilst attending ECEC. Our finding highlights that educator physical activity-related behavior and practices can be improved via a professional development intervention. Our finding also aligns with research that looked at parental support behaviors to improve children’s physical activity. Rhodes et al. [29] showed that parents who were given the resources to plan and support physical activity for their family reported higher family physical activity compared to the control group. Therefore, educator and caregiver’s perceived physical activity practices are an important influencer of preschool children’s physical activity behavior.

Some educator physical activity behaviors may not have had significant opportunity to change as a result of the professional development program due to the baseline values already being high and thus there being little room for further improvement (e.g., importance of physical activity to educators; identified motivation for providing physical activities; perceived time set aside for children to participate in physical activity). However, three educator physical activity behaviors that were relatively high at baseline did significantly change post-professional development: Perceived behavioral control over child physical activity support; perceived time set aside for children to participate in FMS/nature-based play activities; number of barriers around providing physical activities (low). This highlights the potential for both professional development programs to significantly and positively impact educator physical activity behaviors. Importantly, baseline levels of educator physical activity behaviors were on average higher in the FMS professional development group and this may explain the reduced number of PA behaviors that significantly improved in this group at follow up.

After adjusting for educators’ age, qualification, experience in the sector and work hours per week, none of the findings retained statistical significance. This highlights that educator age, qualification, experience in the sector and work hours influenced the association between the intervention and the change in educator physical activity-related behaviors and practices. 

We hypothesized that the intervention effect would be less for those educators who were older, more qualified, more experienced and who worked longer hours because their baseline levels of physical activity-related behaviors and practices would be higher and thus less impacted by the intervention. However, our results showed otherwise; educators in the nature play program with higher qualifications showed a significant improvement in perceived behavioral control over support for children’s nature-based play activities, and educators with more experience in the ECEC sector reported a greater increase in intrinsic motivation for providing physical activities. These findings align with past studies examining the relationship between educators’ qualification and children’s physical activity [30,31,32]. In addition, educators in the FMS professional development program with greater ECEC sector experience reported more barriers to providing physical activities. This may partly be explained by educators with more sector experience being able to identify more barriers due to the increased knowledge about how to overcome potential barriers they had learnt via the FMS professional development program. Furthermore, our study found that those working longer hours reported more perceived behavioral control over support for children’s FMS activities. This suggests that educators who spend longer hours at the center per week were able to spend more time with the children and encourage or participate in FMS related activities with the children. Overall our findings show that ECEC physical activity based professional development programs appear to be more effective for those educators who are more qualified and more experienced. Future ECEC professional development programs should focus on better support to upskill those with lower qualifications and less experience in the ECEC sector.

## 5. Strengths and Limitations

To date most ECEC intervention studies have focused on children and measured children’s physical activity levels as the outcome of interest [33]. Our study is novel in that we looked at the influence of two physical activity professional development programs from the educators’ perspective. A strength of this study was the use of a practical easy to complete 10-min survey to measure educator physical activity-related behaviors and practices before and after the professional development program. Furthermore, there was a three month period between pre and post data collection, which minimized the novelty effect. To our knowledge, this is the first study to pilot a Nature play professional development program to improve educators’ nature play and physical activity behaviors and practices. 

A limitation of this study was that it was a single arm pre-post design so it was not possible to compare the intervention group with a control group. All data were self-reported by ECEC educators which may be subject to response bias. This was a pilot intervention study and future pragmatic studies should include a larger sample size, a comparison group, and a mixed observational approach. Our results may also be subject to selection bias, since all ECEC centers self-selected into the professional development programs.

As this was a pilot intervention aimed at ECEC educators we did not examine the impact of the professional development programs on preschool children’s physical activity. Finally, not all ECEC educators completed both the baseline and post-professional development program survey. This was a result of the high educator turn-over rate at centers which the study team had no control over. 

## 6. Conclusions

Both the Nature play and FMS professional development programs were effective in improving educators’ self-efficacy to engage children in physical activity. The nature play professional development program was also effective in improving educator perceived behavioral control over support for children’s general and nature base play activities. Moreover, these physical activity based professional development programs were most effective for ECEC educators with higher qualifications and more experience in the sector. These findings can be used to further develop these two professional development programs and implement them at scale. Both professional development programs show promise for being an effective and sustainable strategy for improving preschool children’s physical activity in ECEC centers. Larger pragmatic trials are required to confirm the impact of these professional development programs on educators and preschool children’s physical activity behavior.

## Figures and Tables

**Table 1:** Characteristics of study sample.

	Nature Play PD Group			FMS PD Group			Between Group Differences at Baseline	Between Group Differences at Post PD
	Baseline (N = 84)	Post PD (N = 62)	Within Group Differences	Baseline (N = 64)	Post PD (N = 41)	Within Group Differences		
	% or Mean (SD)	% or Mean (SD)	F (*p* Value)	% or Mean (SD)	% or Mean (SD)	F (*p* Value)	F (*p* Value)	F (*p* Value)
Gender (female)	100%	100%		100%	100%			
Mean age	37.0 (9.42)	37.0 (9.00)	−0.08 (0.94)	34.98 (9.59)	34.15 (10.33)	−0.10 (0.92)	−1.10 (0.27)	−1.63 (0.10)
Experience in ECEC sector (months)	133.33 (110.56)	126.71 (101.59)	−0.24 (0.81)	103.29 (73.03)	96.22 (59.39)	−0.22 (0.82)	−0.92 (0.36)	−0.73 (0.46)
Working hours per week at center	33.66 (8.85)	34.64 (8.91)	−0.67 (0.50)	33.37 (7.04)	33.11 (8.05)	−0.51 (0.61)	−0.40 (0.69)	−0.61 (0.54)
Highest education level			−0.31 (0.76)			−0.12 (0.90)	−0.12 (0.90)	−0.07 (0.95)
-Secondary or less	11.3%	9.8%		14.1%	17.1%			
-Trade/apprenticeship/certificate	13.8%	13.1%		14.1%	9.8%			
-Diploma	50.0%	54.1%		40.6%	46.3%			
-Bachelor degree or higher	23.8%	23.0%		28.2%	21.9%			

In the Nature play group, 73.8% ECEC educators completed both surveys. In the FMS group, 53.9% ECEC educators completed both surveys.

**Table 2:** Difference between baseline and post-professional development (PD) results using repeated measures general linear models.

	Nature Play PD Group				FMS PD Group			
	Baseline Mean	Post PD Mean	*p* Value ^1^	*p* Value ^2^	Baseline Mean	Post PD Mean	*p* Value ^1^	*p* Value ^2^
Importance of physical activity to educators ^3^	5.68 (1.68)	5.85 (1.40)	0.91	0.99	5.99 (1.49)	5.88 (1.75)	0.87	0.45
Educator self-efficacy to engage children to be active in general ^4^	3.84 (0.63)	4.10 (0.61)	0.02 *	0.45	3.90 (0.68)	4.06 (0.63)	0.32	0.79
Educator motivations to engage children in physical activities ^3^	4.73 (0.85)	4.68 (0.72)	0.12	0.77	4.74 (0.89)	4.70 (0.79)	0.65	0.56
Intrinsic motivation for providing physical activities ^3^	5.45 (1.04)	5.52 (1.23)	0.68	0.42	5.44 (1.22)	5.56 (1.19)	0.31	0.99
Identified motivation for providing physical activities ^3^	6.03 (0.92)	6.08 (0.86)	0.53	0.82	5.97 (0.97)	6.11 (0.78)	0.15	0.22
Introjected regulation for providing physical activities ^3^	3.66 (1.76)	3.55 (1.73)	0.54	0.92	3.71 (1.81)	3.65 (1.86)	0.63	0.37
External regulation for providing physical activities ^3^	2.94 (1.84)	3.08 (1.84)	0.86	0.45	3.25 (1.94)	3.04 (2.15)	0.47	0.91
Amotivation for providing physical activities ^3^	1.60 (1.14)	1.99 (1.69)	0.06	0.25	1.92 (1.66)	2.04 (1.85)	0.97	0.57
Barriers around providing physical activities (No. of barriers) ^4^	0.20 (0.24)	0.12 (0.22)	0.02*	0.52	0.20 (0.21)	0.33 (0.35)	0.01 *	0.57
Educator self-efficacy to engage children to be active in FMS/nature-based play activities ^5^	3.65 (0.71)	3.98 (0.75)	0.01*	0.12	3.84 (0.83)	4.10 (0.62)	0.03 *	0.99
Perceived behavioral control over child physical activity support (%) ^6^	95.0	100.0	0.00*	0.77	91.9	81.6	0.10	0.11
Perceived time set aside for children to participate in physical activity (%) ^6^	98.8	96.7	0.85	0.69	96.9	96.0	0.39	0.78
Perceived regulation of supportive behaviors for physical activities (%) ^6^	54.2	59.1	0.07	0.74	50.8	54.0	0.99	0.27
Perceived behavioral control over support for children’s FMS/Nature play (%) ^6^	54.2	64.5	0.01*	0.24	61.8	81.6	0.21	0.11
Perceived time set aside for children to participate in FMS/ nature-based play activities (%) ^6^	81.0	90.2	0.01*	0.271	96.8	94.0	0.53	0.60
Perceived regulation of supportive behaviors for FMS/ nature-based play activities (%) ^6^	54.3	64.3	0.07	0.738	50.8	54.0	0.80	0.27

* *p* < 0.05. ^1^ Unadjusted Wilcoxon’s p value. ^2^ Adjusted for educator’s age, qualification, experience in sector and working hours per week at the center. ^3^ Range = 1 to 7. ^4^ Range = 0 to 1. ^5^ Range = 1 to 5. ^6^ High frequency = a response >3.49 on a five-point Likert scale (0 = Never or rarely, 5 = Daily).

**Table 3:** Effect size of educator socio-demographic variables.

	Nature Play PD Group				FMS PD Group			
	ηp^2^				ηp^2^			
	Age only	Qualification Only	Work Hours Only	Work Experience Only	Age Only	Qualification Only	Work Hours Only	Work Experience Only
Importance of physical activity to educators ^1^	0.10	0.02		0.02		0.12	0.04	
Educator self-efficacy to engage children to be active in general ^2^	0.03			0.03			0.03	0.04
Educator motivations to engage children in physical activities ^1^	0.03	0.02		0.03				
Intrinsic motivation for providing physical activities ^1^	0.09		0.03	0.25 *				0.06
Identified motivation for providing physical activities ^1^					0.03	0.07		0.02
Introjected regulation for providing physical activities ^1^				0.04			0.04	
External regulation for providing physical activities ^1^		0.02				0.02		0.02
Amotivation for providing physical activities ^1^	0.03	0.03				0.09	0.07	0.13
Barriers around providing physical activities (No. of barriers) ^2^	0.02				0.02	0.06	0.04	0.04 *
Educator self-efficacy to engage children to be active in FMS/nature-based play activities ^3^	0.04	0.04	0.02		0.02	0.04	0.13	
Perceived behavioral control over child physical activity support (%) ^4^		0.04						
Perceived time set aside for children to participate in physical activity (%) ^4^		0.04			0.02			0.04
Perceived regulation of supportive behaviors for physical activities (%) ^4^	0.02						0.04	
Perceived behavioral control over support for children’s FMS/nature play (%) ^4^	0.02	0.05 *					0.13 *	
Perceived time set aside for children to participate in FMS/ nature-based play activities (%) ^4^	- -	0.02						
Perceived regulation of supportive behaviors for FMS/ nature-based play activities (%) ^4^	0.02	0.04			0.64		0.04	

* *p* < 0.05; ηp2 ≤ 0.01; ^1^ Range= 1 to 7; ^2^ Range = 0 to 1; ^3^ Range = 1 to 5; ^4^ High frequency = a response > 3.49 on a five-point Likert scale (0 = Never or rarely, 5 = Daily).
